# Phosphoproteomics data classify hematological cancer cell lines according to tumor type and sensitivity to kinase inhibitors

**DOI:** 10.1186/gb-2013-14-4-r37

**Published:** 2013-04-29

**Authors:** Pedro Casado, Maria P Alcolea, Francesco Iorio, Juan-Carlos Rodríguez-Prados, Bart Vanhaesebroeck, Julio Saez-Rodriguez, Simon Joel, Pedro R Cutillas

**Affiliations:** 1 Analytical Signalling Group, Centre for Cell Signalling, Barts Cancer Institute, Queen Mary University of London, Charterhouse Square, London EC1B 6BQ, UK; 2 European Bioinformatics Institute, EMBL-EBI, Wellcome Trust Genome Campus - Cambridge CB10 1SD, UK; 3 Cancer Genome Project, Wellcome Trust Sanger Institute, Wellcome Trust Genome Campus - Cambridge CB10 1SD, UK; 4 Cell Signalling Group, Centre for Cell Signalling, Barts Cancer Institute, Queen Mary University of London, Charterhouse Square, London EC1B 6BQ, UK; 5 Centre for Haemato-Oncology, Barts Cancer Institute, Queen Mary University of London, Charterhouse Square, London EC1B 6BQ, UK; 6 Current address: MRC Clinical Sciences Centre, Faculty of Medicine, Imperial College London, Hammersmith Hospital Campus, Du Cane Road, London, W12 0NN, UK

## Abstract

**Background:**

Tumor classification based on their predicted responses to kinase inhibitors is a major goal for advancing targeted personalized therapies. Here, we used a phosphoproteomic approach to investigate biological heterogeneity across hematological cancer cell lines including acute myeloid leukemia, lymphoma, and multiple myeloma.

**Results:**

Mass spectrometry was used to quantify 2,000 phosphorylation sites across three acute myeloid leukemia, three lymphoma, and three multiple myeloma cell lines in six biological replicates. The intensities of the phosphorylation sites grouped these cancer cell lines according to their tumor type. In addition, a phosphoproteomic analysis of seven acute myeloid leukemia cell lines revealed a battery of phosphorylation sites whose combined intensities correlated with the growth-inhibitory responses to three kinase inhibitors with remarkable correlation coefficients and fold changes (> 100 between the most resistant and sensitive cells). Modeling based on regression analysis indicated that a subset of phosphorylation sites could be used to predict response to the tested drugs. Quantitative analysis of phosphorylation motifs indicated that resistant and sensitive cells differed in their patterns of kinase activities, but, interestingly, phosphorylations correlating with responses were not on members of the pathway being targeted; instead, these mainly were on parallel kinase pathways.

**Conclusion:**

This study reveals that the information on kinase activation encoded in phosphoproteomics data correlates remarkably well with the phenotypic responses of cancer cells to compounds that target kinase signaling and could be useful for the identification of novel markers of resistance or sensitivity to drugs that target the signaling network.

## Background

Hematologic malignancies are a group of neoplastic diseases that originate from the transformation of bone marrow-derived cells. This group, which includes leukemias, lymphomas, and myelomas, is extraordinarily heterogeneous, which reflects the complexity of normal hematopoiesis and the immune system [1]. Although gene expression signatures can be used to classify malignancies into subgroups [2-4], a system-level understanding of the biochemical pathways (both signaling and metabolic) responsible for tumor phenotypes requires knowledge of signaling pathway activity, information that cannot be provided by measuring mRNA or protein expression alone [5,6], as enzyme expression does not necessarily correlate with pathway activity [7].

Essentially all cancers are driven by deregulation of protein kinase cascades downstream of growth factor, antigen, and G protein-coupled receptors [8]. Consequently, several kinase inhibitors that block cell transduction pathways overactive in cancer are already in the clinic while others are undergoing pre-clinical or clinical development. However, although clinical impact is observed in some patients, many patients do not respond to these therapies or subsequently develop resistance [9,10]. The use of predictive biomarkers, or 'companion diagnostics', is therefore important in individualizing such targeted agents [11]. While the activity of the target kinase can in some instances predict response [12], this is not always the case, as the activity of parallel pathways in the network can contribute to resistance [13,14]. It could therefore be envisaged that the analysis of kinase signaling without a preconception of the pathways that may be active could be advantageous in predicting responses to kinase inhibitors.

Phosphorylation is a posttranslational modification regulated by the activity of kinases and phosphatases. By definition, each phosphorylation site is the result of a kinase/phosphatase reaction pair. Changes in phosphorylation status can alter many aspects of protein biology, including their localization, protein-protein interactions, stability, and enzymatic activity [15]. Although the information coded by phosphorylation patterns has not been completely deciphered, many phosphorylation sites can be associated with the activity of a specific protein kinase and thereby classified into signaling pathways [16-18]. Thus, global analysis of protein phosphorylation using quantitative techniques may in principle be translated into knowledge of the activation status of signaling pathways. This information, in turn, could be used to rationalize how the wiring of the kinase network contributes to the phenotypic characteristics of different tumors, such as aggressiveness, metastatic potential, and sensitivity to therapy.

The application of new proteomic techniques for phosphopeptide quantification is contributing to an improved understanding of cancer cell biology [19-23]. Several techniques for quantitative proteomics have been developed; these can be divided into those that require labeling of proteins with stable isotopes (for example, SILAC and iTRAQ) and those that do not require labeling [24,25]. Approaches based on labeling techniques usually detect a larger number of phosphopeptides than those based on label-free approachesbecause labeling techniques are compatible with extensive fractionation prior to mass spectrometry analysis. However, because of the time-consuming nature of such analyses, studies based on labeling techniques normally compare a small number of samples with no (or very few) biological replicates, a feature that limit the statistical significance of the results. Therefore there is a trade-off between the number of peptide/proteins identified and samples that can be compared in a study. Label-free approaches are preferred when the aim is to compare large sample numbers and replicates [26,27] even though the penetrance of the approach may not be as large as when using techniques that allow extensive fractionation before mass spectrometry analysis.

In the current study, label-free mass spectrometry (MS) was first used to analyze the phosphoproteomes of nine different hematological cancer cell lines. Unsupervised analysis of the data based on principal component analysis (PCA) and hierarchical clustering classified these cell lines according to their pathological origin, namely acute myeloid leukemia (AML), lymphoma, or multiple myeloma. Through a lasso linear regression analysis we assessed the potential of this data in predicting the level of sensitivity of seven AML to three kinase inhibitors: PI-103 (a PI3K/mTOR inhibitor), MEK-i, and JAK-i. Finally we identified phosphopeptides whose intensities across the cell lines correlated with the sensitivity to three inhibitors. Our results revealed a battery of phosphorylation sites whose intensities strongly correlated with responses of our AML panel to the compounds (with R > 0.9 and > 100-fold difference between most sensitive and most resistant cell line). These data therefore indicate that MS-based quantitative phosphoproteomics has the potential to classify cell lines into distinct subgroups according to their pathological origin and to their sensitivity to drugs that target kinase signaling. Phosphorylation sites that correlated with resistance were enriched in basic and proline-directed motifs, whereas those that correlated with sensitivity were mainly acidic or hydrophobic containing, thus suggesting that the relative activities of basophilic, proline directed, and acidophilic kinases may determine responses to the inhibitors. Therefore, the results of our study indicate that unbiased profiling of phosphorylation has the potential to stratify AML cells based on their responses to signaling inhibitors because these responses may be dependent on the combination of pathways (both target and parallel) active in cells, rather than on the activity of the target kinase/pathway only.

## Results

### Overview of protein phosphorylation in hematological cancer cells

A group of nine different hematological cell lines, consisting of three acute myeloid leukemia (AML), three lymphoma, and three multiple myeloma cell lines (Additional file 1, Table S1), was selected for analysis using a quantitative LC-MS/MS phosphoproteomics workflow summarized in Additional file 2, Figure S1A. This approach to quantify phosphorylation, which involves comparing peak intensities of phosphopeptides calculated as the height and areas of ions extracted from aligned chromatograms, has been independently validated by immunoblotting in our previous work that showed that this methodology can be used to quantify phosphopeptide levels with good precision and accuracy [16,28]. The technique is similar to that used in other laboratories [29-31].

Three biological replicates were analyzed on two separate occasions to give a total of six replicates per cell line, requiring 54 LC-MS/MS runs. The approach led to the identification of 2,050 phosphopeptides in 1,664 proteins (Additional file 3, Dataset 1). Given that phosphopeptides can contain more than one phosphorylation site, in total we identified 2,434 unique phosphorylation sites. Of these, the precise position of the modification was ambiguous for 738 sites. The remaining 1,696 sites (70% of total) were classified according to the amino acid bearing the site of phosphorylation. Additional file 2, Figure S1B shows that 1,254 (74%), 344 (20%), and 98 (6%) of phosphorylation sites were on serine, threonine, and tyrosine, respectively. These results are comparable with the distribution of phosphorylation sites in HeLa carcinoma cells and in platelets [32,33].

In order to further assess the nature of the phosphoproteomes identified in this study, we compared the assigned gene ontologies (GO) of the phosphoproteins with those proteins present in the whole human proteome (obtained from the SwissProt database, Additional file 4, Figure S2). Each protein was classified according to the three different domains included in the gene ontology project [34], namely (1) cellular component which describes the subcellular localization of the identified protein (or its extracellular environment); (2) biological process which indicates the biological function to which the gene product contributes; and (3) molecular function which describes the elemental activities of a gene product at a molecular level [34]. The distributions of these domains in our phosphoprotein database were compared to those in the SwissProt database (which lists all the known gene products and can thus be considered to represent the whole human proteome). The data show that phosphoproteins were on the whole not biased towards any GO, in that the subcellular distribution of phosphoproteins identified were similar to those reported in the SwissProt database (Additional file 4, Figure S2A). The minor differences in the proportion of membrane proteins could be explained by the difficulty in solubilizing membrane proteins with buffers compatible with the rest of the workflow. We also observed that phosphoproteins located in the cytosol/cytoplasm and those with roles in translation, cell cycle regulation, and proliferation were well represented relative to the whole proteome (Additional file 4, Figure S2A and S2B). Other well represented phosphoproteins include those with protein kinase activity and other enzymes (Additional file 4, Figure S2C).

### Phosphoproteomics classified hematological cancer cells according to pathology

**Figure 1:**
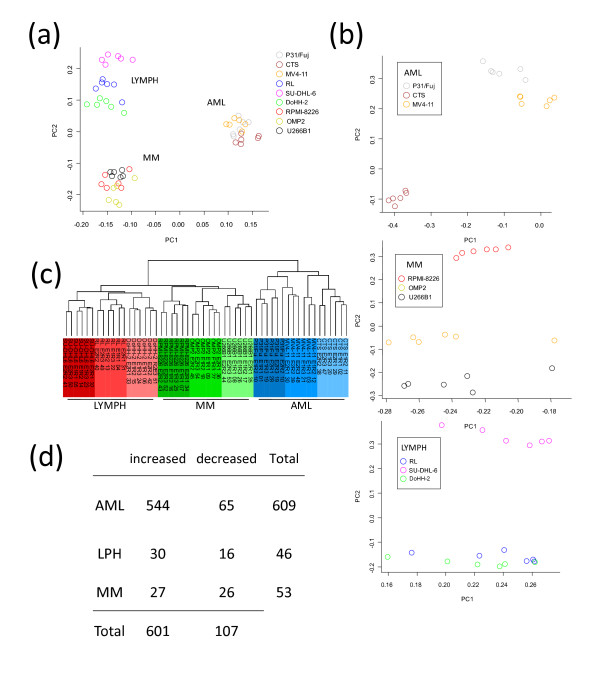
**Phosphoproteomics classify hematological cell lines according to their tissue origin**. Phosphopeptides identified by LC-MS/MS with Mascot and quantified with Pescal in nine hematological cell lines were analyzed by two different clustering tools. (a) Principal component analysis (PCA) of all the cell lines based on the analysis of the most intense 1,500 phosphopeptides. (b) Independent PCA plots of cells belonging to the same pathological group. (c) Unsupervised hierarchical clustering using Kendall's tau coefficient to measure association between peptides and arrays and complete linkage clustering to calculate distances between clusters; AML samples are highlighted in red, lymphoma in blue, and multiple myeloma in green. Each replicate is shown separately and the cell line number is followed by the order in which the samples were analyzed by LC-MS/MS. (d) Summary of phosphopeptides differentially regulated in AML, lymphoma, and multiple myeloma cell lines with two-fold difference over mean expression and *P *< 0.05 after t-test and FDR multiple test correction.

We next asked whether quantitative phosphoproteomics could be used to classify hematological cell lines according to their pathological origin. Normalized peptide intensities were used to calculate the overall fold difference (relative to the mean value across all samples) for each phosphopeptide identified and quantified in our study. These data were then subjected to principal component analysis (PCA). When analyzing the nine cell lines together, inspection of the PCA outputs (Figure 1A) revealed that principal component 1 (PC1) produced two clearly separated groups, one containing AML cell lines and another containing lymphoma and multiple myeloma cell lines, while principal component 2 (PC2) separated cells derived from lymphoma from those that originated from multiple myeloma (Figure 1A). We also analyzed the phosphorylation data of each cell line in three separate PCA plots corresponding to the three different cancer types. Only data on the cell lines of each different malignancy were included in each particular PCA plot. These data, summarized in Figure 1B, revealed that cell lines within a given disease could also be separated based on their phosphoprotein content, indicating global differences in phosphorylation between cells of the same pathology with the exception of RL and DoHH2 lymphoma cell lines which could not be separated by PCA (Figure 1B).

The ability of phosphoproteomics to classify hematological cell lines was also assessed by unsupervised hierarchical clustering. This analysis also classified the samples into three main groups corresponding to AML, lymphoma, and multiple myeloma, and clustered replicates for each cell line within those groups. Interestingly lymphoma and multiple myeloma cells were clustered together, consistent with AML cells being the most differentiated group (Figure 1C). This is consistent with the data obtained by PCA (Figure 1A). Taken together, the data in Figure 1 indicate that although there are differences in global phosphoprotein abundance within cell lines of the same pathology, the differences in the phosphoproteomes of hematological cancer cells are greater across pathology groups than within a given disease.

Stringent statistical analysis (as indicated in materials and methods) identified 609 phosphopeptides differentially regulated in the AML cell lines, of which 544 showed increased phosphorylation and 65 decreased phosphorylation, 46 were different in lymphoma cells (30 increased and 16 decreased) and 53 in multiple myeloma (27 increased and 26 decreased) (Figure 1D). Representative examples of phosphopeptides increased or decreased in AML, lymphoma, and multiple myeloma cells are shown in Additional file 5, Figure S3. Taken together, these data show that label-free quantitative phosphoproteomic data can be used to reproducibly classify hematological cancer cell lines according to their pathological origin.

### Phosphorylation patterns in hematological cancer cells associated with sensitivity/resistance to kinase inhibitors

**Figure 2:**
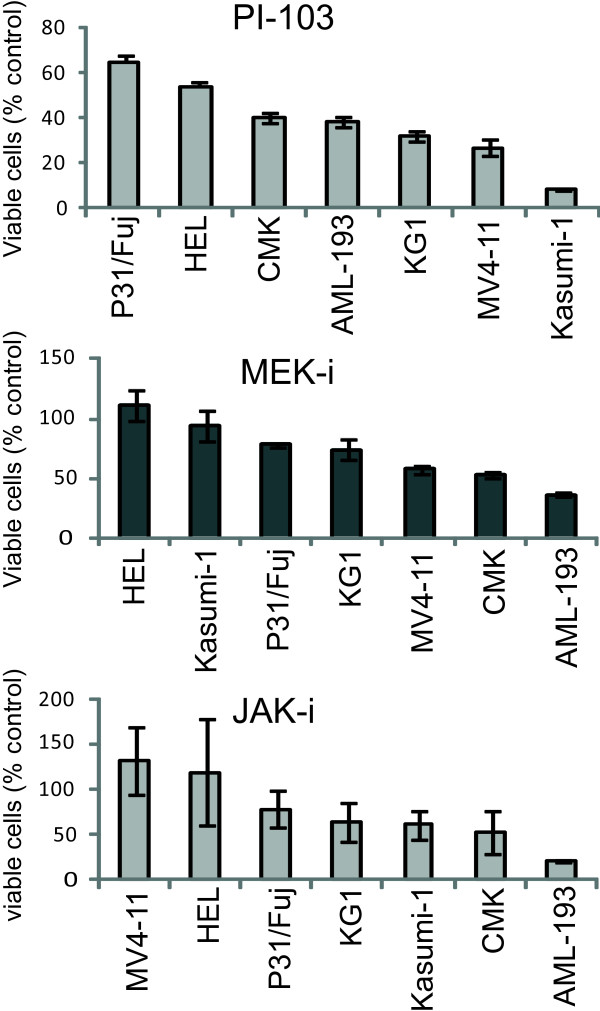
**Responses of hematological cell lines to kinase inhibitors**. AML cells were exposed to 1 μM PI-103, 10 μM MEK-i, or 1 μM JAK-i for 72 h and viability measured by MTS and expressed as percentage to DMSO (control) treated cells. Cell lines were then ranked according to their responses to the inhibitors. Values are mean ± SD (*n *= 4).

In order to assess whether phosphoproteomicscould also be used to classify cells according to their responses to kinase inhibitor treatment, we compared phosphorylation patterns with the sensitivity of a panel of seven AML cell lines (Additional file 1, Table S2) to a PI3K/mTOR inhibitor (PI-103), a MEK-inhibitor (MEK-i), and a JAK-inhibitor (JAK-i). Cell lines were ranked according to the percentage reduction in MTS signals when incubated with 1 μM PI-103, 1 μM JAK-i, or 10 μM MEK-i (Figure 2). MTS measures mitochondrial redox activity, a parameter that often correlates with cell viability. In our assay a reduction in MTS signal as a function of compound treatment may be taken to indicate a reduction in the number of viable cells relative to the control as a combined action of the drugs in inhibiting proliferation and inducing cell death.

We then performed phosphoproteomics analysis on these cell lines (the data are shown in Additional file 6, Dataset 2). These experiments were performed in three independent cell cultures per cell line and further data analysis was based on phosphopeptide intensities from individual replicates rather than the averages values of the three replicates.

### Assessing the potential of phosphoproteomics data in predicting drug responses

We assessed the ability of phosphoproteomics data in predicting the drug sensitivity profiles of our panel of cell lines against the three tested inhibitors through a 'leave-one-cell-line-out' (LOCLO) approach based on lasso regression [55], detailed in the methods section. For each of the three tested drugs, we trained seven different models by leaving out the samples corresponding to each of the seven cell lines in turn, and composing each time a test set with them. The trained models were finally used to make predictions on the test set.

Each training phase was composed by a three-fold cross validation estimation of the lasso shrinkage parameter (involving 18 of the corresponding training set only) followed by an optimization phase of the regressor coefficients (on the training set) and was repeated 20 times. Finally an average trained model was assembled across these 20 iterations and used to predict drug-responses for the corresponding test set.

**Figure 3:**
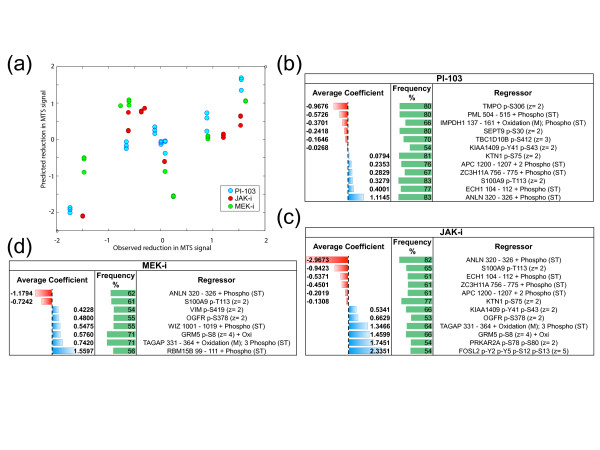
**Lasso regression results**. (a) Scatter plot of the viability scores predicted in the 'leave-one-cell-line-out' iterations for PI-103 (blue), JAK-i (red), and MEK-i (green) *versus *the observed ones. For comparability, all the scores were normalized in order to have zero mean, and unitary standard deviations. Predicted scores were normalized together with the training-set prediction in the corresponding iteration while observed scores were normalized drug-wisely. The inserts report average non-null coefficients and average non-null coefficient rate in the final descriptive models for PI-103 (b), JAK-i (c), and MEK-i (d) for phosphopeptides whose rate is > 50% (that is, sufficiently stably included in the optimal models).

A scatter plot of the predicted viability scores for each of the test set *versus *the observed ones is depicted in Figure 3A. To make results comparable in this plot, test set predictions were normalized together with the corresponding training set predictions and for the same reason, all the observed viability scores were normalized drug-wisely. A remarkably high and statistically significant values of Pearson correlation coefficient (R) were measured between observed/predicted viability scores summarizing response to treatment with PI-103 (R = 0.94, *P *value = 1.15 × 10^-10^) and JAK-i (R = 0.84, *P *value = 1.53 × 10^-6^). When considered together, the observed/predicted viability scores for these two drugs was still high and significant (R = 0.72, *P *value = 8.77 × 10^-8^) while the overall correlation considering all the three drugs was weaker (R = 0.53, *P *value = 9.86 × 10^-6^) and no statistically significant R was observed on the MEK-i scores alone. Scatter plots for individual drugs, with cell line identifiers and all the R scores and *P *values are provided in Additional file 7, Figure 4.

**Figure 4:**
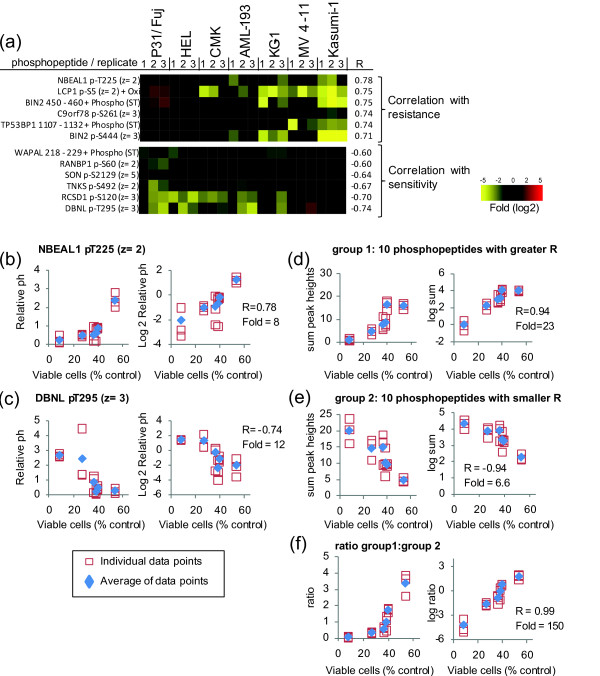
**Correlating responses to PI-103 with phosphoproteomics data**. The normalized intensities of phosphopeptides quantified across seven AML cell lines were correlated with their responses to the inhibition of proliferation after exposure to 1 μM PI-103, as measured by cell viability. (a) Phosphopeptides showing greater correlation with resistance or sensitivity to PI-103. (b) Relationship between normalized intensity of phosphorylated NBEAL1 and responses to PI-103. (c) Relationship between normalized intensity of phosphorylated DBNL and responses to PI-103. (d) Correlation of the sum of the intensities showing greater correlation with resistance. (e) Correlation of the sum of the intensities showing greater correlation with sensitivity. (f) Correlation of the ratio between phosphopeptide intensities showing greater correlation to resistance relative to those showing greater correlation with sensitivity. In each case, R refers to the Pearson correlation coefficient of the log2 transformed data; fold refers to the division of the intensities of the peptide group increased in the most resistant cell line by that of the most sensitive cell line; open squares are individual data points of three biological replicates; blue diamonds are the averages of the three replicates.

Finally, for each of the three tested drugs, we determined a final descriptive model by pooling together (as detailed in the method section) all the models generated in the corresponding LOCLO iterations. These final models, provided in Additional file 8, Dataset 3, contained 33, 32, and 41 phosphopeptides (respectively for PI-103, JAK-i, and MEK-i) that were contained in at least one of the final models generated during the LOCLO iterations together with their coefficient averaged across the iteration and the frequency through which this was different from zero (that is, percentage of LOCLO iterations in which the phosphopeptide was included in the model).

In Figure 3 (B, C, and 3D) we reported excerpts of the final descriptive models including only regressors whose inclusion frequency is > 50% (sufficiently stably included).

Taken together these results suggest that phosphoproteomics data have the potential to predict responses to the analyzed compounds in cell line models and that a relatively small core-set of phosphopeptides is strongly associated to viability scores.

### Identification of subsets of phosphorylation sites that correlate with sensitivity

To complement the analysis based on LASSO, we also performed a correlation study to identify further phosphopeptides associated with responses to the three inhibitors. Figure 4A illustrates phosphopeptide intensities showing stronger associations with resistance or with sensitivity to PI-103. We observed that the correlations with responses were not always linear but instead often followed an exponential trend. This is illustrated in Figure 4B and 4C (left graphs) for phosphopeptides derived from NBEAL pT225 and DBNL pT295 which showed the greater correlation with resistance or sensitivity, respectively. Expressing normalized peak heights as a function of cell viability in semi-logarithmic scale produced a linear relationship between viability and phosphopeptide intensities (Figure 4B and 4C, right graphs) with R = 0.78 for NBEAL and R = -0.74 for DBNL phosphopeptides, consistent with the existence of an exponential relationship between viability and phosphopeptide intensities for these peptides.

We also investigated whether a combination of several phosphopeptides could be used to find associations between our panel of AML cells and their response to PI-103. As Figure 4D shows, the added intensities of the 10 phosphopeptides with greater correlation with the responses to PI-103 (denoted as group 1 in Figure 4D) showed a very strong correlation (R = 0.94) with the viability of cells exposed to PI-103. As with the behavior of single phosphopeptides, the correlation was more linear in semi-log than in linear scale (Figure 4D). The sum of the phosphopeptides with greater correlation with sensitivity (denoted as group 2 in Figure 4E) also showed a linear relationship with cell viability in semi-log scale (R = -0.94, Figure 4E). Remarkably, the ratio of phosphopeptide intensities that correlate with resistance (group 1) to those that correlate with sensitivity (group 2) showed a Pearson score of R = 0.99 with viability (Figure 4F). The differences in combined phosphopeptide intensities between the most resistant and most sensitive cell lines was 150-fold for the group 1:group 2 ratio (Figure 4F) compared to just 23-fold for group 1 (Figure 4D), indicating that the ratio of phosphopeptide intensities that correlate with resistance relative to those that correlate with sensitivity could be a strong classifier for the stratification of AML cells based on the likelihood that they respond to PI-103.

**Figure 5:**
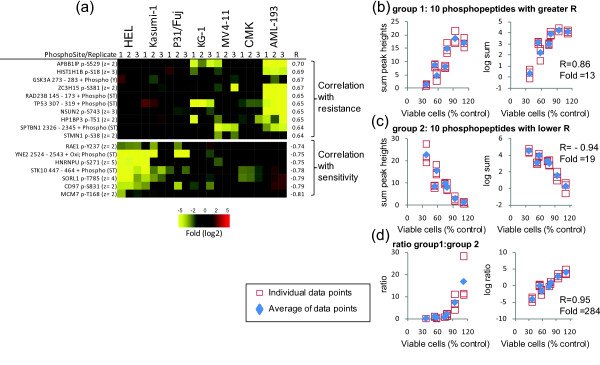
**Correlating responses to MEK-i with protein phosphorylation data**. The normalized intensities of phosphopeptides quantified across seven AML cells were correlated with the responses to the inhibition of proliferation of cells exposed to 10 μM MEK-i, as measured by cell viability. (a) Phosphopeptides showing greater correlation with resistance or sensitivity to MEK-i. (b) Correlation of the sum of the intensities showing greater correlation with resistance. (c) Correlation of the sum of the intensities showing greater correlation with sensitivity. (d) Correlation of the ratio between phosphopeptide intensities showing greater correlation to resistance relative to those showing greater correlation with sensitivity. In each case, R refers to the Pearson correlation coefficient of the log2 transformed data; fold refers to the division of the intensities of the peptide group increased in the most resistant cell line by that of the most sensitive cell line; open squares are individual data points of three biological replicates; blue diamonds are the averages of the three replicates.

**Figure 6:**
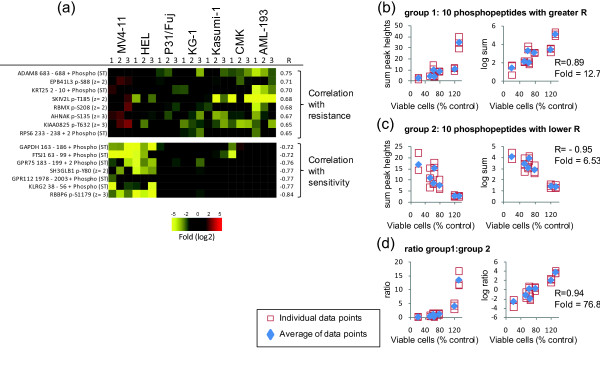
**Correlating responses to JAK-i with global protein phosphorylation**. The normalized intensities of phosphopeptides quantified across seven AML cells were correlated with the responses to the inhibition of proliferation of cells exposed to 1 μM JAK-i, as measured by cell viability. (a) Phosphopeptides showing greater correlation with resistance or sensitivity to JAK-i. (b) Correlation of the sum of the intensities showing greater correlation with resistance. (c) Correlation of the sum of the intensities showing greater correlation with sensitivity. (d) Correlation of the ratio between phosphopeptide intensities showing greater correlation to resistance relative to those showing greater correlation with sensitivity. In each case, R refers to the Pearson correlation coefficient of the log2 transformed data; fold refers to the division of the intensities of the peptide group increased in the most resistant cell line by that of the most sensitive cell line; open squares are individual data points of three biological replicates; blue diamonds are the averages of the three replicates.

Phosphopeptide intensities were also correlated with the responses to MEK-i and JAK-i. Phosphopeptides showing the stronger association with resistance or sensitivity to MEK-i are shown in Figure 5A. The added intensities of phosphopeptides that correlated with resistance (group 1) or sensitivity (group 2) are shown in Figure 5B and 5C, respectively. As with the analysis of phosphopeptides correlating with responses to PI-103, the ratio of group 1 to group 2 showed a greater linear correlation (R = 0.95) and fold difference (284) between the most sensitive and resistant cells to MEK-i (Figure 5D) than when considering either group alone (Figures 5B and 5C). Similar results were obtained when the phosphoproteomes of our AML cell line panel were correlated with resistance/sensitivity to JAK-i. Figure 6A shows the phosphopeptides with best correlation with the response to this compound. While the combined intensities of the phosphopeptides that correlated with resistance (group 1, Figure 6B) or sensitivity (group 2, Figure 6C) showed good correlation with viability (R = 0.89 and -0.95, respectively), the ratio of group 1 to group 2 phosphopeptide intensities showed the greater fold difference between the most resistant and sensitive cell lines to JAK-i (fold = 76.8), along with a strong linear correlation of the semi-log transformed data (R = 0.94, Figure 6D).

To investigate whether phosphopeptides associated with resistance/sensitivity in AML cells (Figures 4 to 6) would also be associated with responses in other cell types, we assessed whether these potential markers of sensitivity would also correlate in lymphoma and multiple myeloma cells shown in Figure 1. This analysis was undertook with the view that not all sites associated with responses in AML may be associated with the same parameters in lymphoma or multiple myeloma because of the marked differences in phosphorylation across these malignancies (Figure 1). First, sensitivity of the lymphoma and multiple myeloma cells shown in Figure 1 to PI103, JAK-i, and MEK-i was determined using MTS (Additional file 9, Figure S5A). These values of sensitivity were then correlated withthe phosphorylation patterns of the corresponding peptides previously found associated to the inhibitors in the AML cells. Although not all markers of sensitivity found for AML were also associated with responses in lymphoma or multiple myeloma, we found that the phosphorylation signals of PML p-S506 and MARCKS p-S170 also positively correlate, with viability after treatment with PI-103 in our set of lymphoma and multiple myeloma cells (R = 0.72 and 0.59, respectively) (Additional file 9, Figure S5B). Similarly, phosphorylation signals on KRT25 p-S7 and SKIV2L p-T186 positively correlated with viability after treatment with JAK-i (R = 0.63 and 0.78, respectively) while the phosphorylation of HNRNPU p-272 negative correlated with the viability after treatment with MEK-i (R = 0.97 and 0.82) (Additional file 9, Figure S5C and S5D). Thus these data (Additional file 9, Figure S5) show that phosphorylation markers may be able to predict responses to kinase inhibitors across different diseases despite their marked differences in basal phosphorylation (Figure 1).

### Pathways and ontologies associated with responses to kinase inhibitors

In order to investigate pathways represented in phosphopeptide sets associated with the responses of our AML cell line panel to kinase inhibitors, we performed a bioinformatics analysis using DAVID pathway analysis tools [35]. This analysis was based on phosphopeptides correlating with responses with R > 0.45 or R < -0.45. Phosphopeptides that correlated with the responses to MEK-i and JAK-i were present in proteins with diverse functions, including mRNA splicing, transcription and nuclear proteins. PKC pathway members were significantly enriched (*P *< 0.001) in the dataset of phosphorylation sites that correlated with the resistance to PI-103 (Additional file 10, Figure S6A). Ten phosphorylation sites on kinases, six of which are Ser/Thr protein kinases, were also found to correlate with resistance (Additional file 10, Figure S6B). Phosphorylation sites on proteins involved in transcription were well represented in the dataset that correlated with sensitivity to PI-103 (Additional file 10, Figure S6C, although the enrichment was not statistically significant, *P*= 0.2) or with resistance (Additional file 10, Figure S6D).

**Figure 7:**
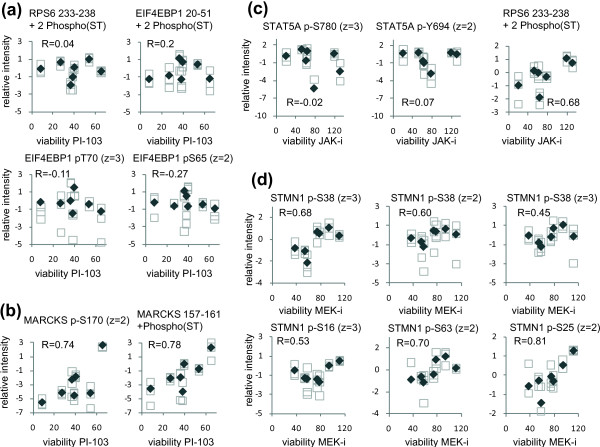
**Correlation of known PI3K, JAK and MEK pathway markers with responses to kinase inhibitors**. (a) Correlation of phosphorylated peptides containing phosphorylation sites known to be downstream of PI3K/mTOR with sensitivity to PI-103. (b) Correlation of phosphorylated peptides on MARCKS, known to contain sites phosphorylated by PKCs, with responses to PI-103. (c) Correlation of phosphorylation sites on STAT5A, a known JAK site, and ribosomal S6 (RPS6 gene product) with sensitivity to JAK-i. (d). Correlation of several sites on stathmin (STMN1 gene product) with the sensitivity to MEK-i. Open squares: individual data points; filled diamonds: averages of individual data points.

To assess whether phosphorylation sites on known members of PI3K, MEK, and JAK pathways would correlate with the responses of our AML cell line panel to the kinase inhibitors tested, we also specifically considered phosphorylation sites that, based on the literature, are known to be downstream of these kinases. Phosphorylation sites on 4EBP1 (gene name EIF4EBP1) or Ribosomal S6 (gene name RPS6), which are known to be downstream of PI3K/mTOR(Ref [36]), did not correlate with the responses of our cancer cell panel to the PI3K/mTOR inhibitor PI-103 (Figure 7A). In contrast, it was interesting to observe that phosphorylation sites on two phosphopeptides derived from MARCKS, which are substrates of PKCs (Ref [37]) and which thus provide a measure of PKC activities, correlated (R = 0.74 and 0.78) with the resistance of our AML panel to PI-103 (Figure 7B). These results are in line with those from the pathway analysis shown in Additional file 10, Figure S6A suggesting a correlation between the phosphorylation of PKC pathway members and the resistance to PI-103 and raised the hypothesis that PI-103 resistant cells were using the PKC pathway to proliferate. To explore this possibility, we treated P31/Fuj, HEL, and MV4-11 cells with different combinations of the PI3K/mTOR inhibitor PI-103 and the PKC inhibitor Go6976. We found an additive effect of combining these compounds in the viability of all these cell lines (Additional file 11, Figure S7A). Interestingly, the cell lines with greater concentrations of phosphorylated MARKCS (a marker of PKC activity) were more resistant to the PKC inhibition relative to those with low phospho-MARCKS (Additional file 11, Figure S7B and S7C). These data are in line with previous findings suggesting that the PKC signaling pathway has a role in the proliferation and/or survival of AML cell lines ([28]).

We also found that while the intensities of phosphorylated STAT5A, a marker of JAK activities, did not correlate with the responses of our AML cell panel to JAK-i, the intensities of phosphorylated Ribosomal S6 (a marker of PI3K/mTOR pathway activation [36]) did correlate with resistance to this compound (Figure 7C). As an example of a phosphoprotein that correlated with the responses to MEK-i, Figure 7D shows the correlation of several phosphorylation sites on Stathmin, alongside the viability of cells exposed to this compound. The phosphorylation of S16, S38, and S63 on Stathmin followed the same trend and had a relatively poor correlation with the response to MEK-i (Figure 7D) compared to the phosphorylation of S25, a site that can be phosphorylated by several MAP kinases and cyclin dependent kinases [38,39]. Taken together, the data shown in Figure 7 suggest that the activation of the pathway being targeted did not in general correlate with the response to kinase inhibitors and that phosphorylations that correlate with responses predominantly were on pathways parallel to those being targeted.

### Phosphorylation motifs associated with responses to kinase inhibitors

Phosphoprotein concentrations in cells can be a consequence of the expression level of the phosphoprotein bearing the site of modification as well as of the activity of the kinases and phosphatases acting on these sites. To investigate whether the phosphorylation sites that correlated with resistance or sensitivity in this study were the result of differential kinase activities across the AML cell line panel or these were just a reflection of phosphoprotein gene expression, we performed a further bioinformatics analysis of the data aimed at assessing common phosphorylation motifs in phosphopeptides correlating with the responses to the inhibitors. In this, phosphorylation motifs were obtained from the data using motif-X [40] and from the literature [41]. The normalized intensities of phosphorylation sites bearing these motifs were then averaged and correlations with the viability of cells to each inhibitor recalculated for each of the averaged motif intensities. This analysis was based on phosphopeptides with R > 0.45 or R < -0.45.

**Figure 8:**
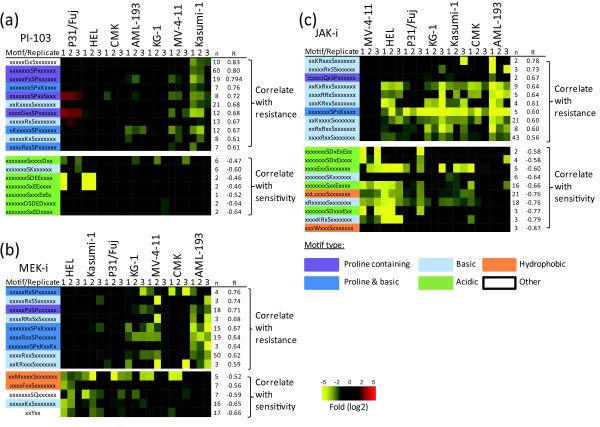
**Phosphorylation motifs that correlate with the responses to kinase inhibitors**. Phosphopeptides showing correlation to the responses to kinase inhibitions were grouped based on these containing specific phosphorylation motifs. The intensities of phosphopeptides in these groups were then averaged and correlated with viability of cells after exposure to PI-103, MEK-i, or JAK-i. Motifs were then ranked according to their correlation to the responses to PI-103 (a), MEK-i (b), or JAK-i (c). Motifs were color-coded based on their type. R, Pearson correlation to semi-log transformed data; n, number of phosphopeptides matched to the named motif.

Figure 8 shows that phosphorylation motifs that correlated with resistance were rich in basic residues and/or had a proline at the carboxyl terminus of the phosphorylated residue. In contrast, phosphorylation motifs that correlated with sensitivity to the inhibitors were predominantly on acidic motifs for PI-103 and JAK-i (Figure 8A and 8C) or they contained a hydrophobic residue at the N terminus for JAK-i and MEK-i (Figure 8B and 8C). These data suggest that basophilic kinases (which include the AGC family protein kinases such as S6K, PKC, PKA, and PKB) and proline directed kinases (which include mTOR, CDKs, and MAP kinases) may be more active in resistant cells (Figure 8), whereas acidophilic kinases (such as casein kinases) and hydrophobic amino acid directed kinases (which include AMPK) may be more active in sensitive cells. These data are consistent with the correlation of PKC activity with resistance to PI-103 inferred from pathway and substrate phosphorylation analyses (Figure 8 and Additional file 9, Figure S5) in that the averaged intensities of 13 peptides containing the preferred motif for PKC (xRxSx) and of eight peptides containing the related xKxSx motif also correlated with the resistance to PI-103 (Figure 8A). Also consistent with the data shown in Figure 7C, suggesting a correlation between S6K activity and the resistance to JAK-i, the combined intensities of 43 phosphopeptides in the context of the xRxxSx motif also correlated with the resistance to this compound (Figure 8C). Of these, eight and nine phosphopeptides were in the context of the RxRxxSx and KxRxxSx motifs, respectively, which are the preferred recognition motifs of S6K and related upstream kinases such as PKB/Akt. Overall, the data in Figure 8 indicate that the correlation of phosphopeptide intensities with resistance and sensitivity to kinase inhibitors is most likely due to differential kinase pathway activation in these cells, rather than just reflecting differences in the expression levels of the phosphoproteins bearing the sites of modification.

## Discussion

Tumor classification informs therapeutic strategies and provides information on prognosis [42]. A classification based on the likely response to a particular therapy may be of particular importance in the use of agents that target protein and lipid kinases in signaling pathways [43]. These enzymes are drug targets deregulated in essentially all cancers [8] but the lack of robust and generally applicable methods to stratify patients for therapies based on kinase inhibitors limit their clinical applicability [11]. DNA sequencing, expression arrays and proteomic signatures have been used both to classify tumors and to indicate the presence of mutation status or expression of genes involved in cancer cell signaling [44-46]. However, protein, gene, or mRNA studies do not necessarily reflect enzyme activity, which ultimately determines the activation status of signaling pathways involved in cancer cell biology [5,6,47,48]. Protein phosphorylation is the result of the activities of kinases present in the pathways that are being targeted by signaling inhibitors and kinase activity is a major determinant in conferring resistance/sensitivity to these compounds. Therefore, it may be argued that a comprehensive analysis of protein phosphorylation might represent an ideal readout to classify tumors based on the likelihood of response to inhibitors that target signaling pathways.

In order to investigate whether phosphoproteomics may be used to classify hematological cancers based on their phenotype, and to provide proof-of-principle of the approach, we analyzed the most abundant phosphoproteomes of nine hematological cancer cell lines. Qualitative analysis of the data based on Gene Ontologies revealed that our analysis was broad and included phosphopeptides from all types of proteins, including those in cell membranes, cytosol, and subcellular compartments. While membrane proteins were slightly less represented than expected, proteins involved in cell cycle regulation, proliferation, and translation, and protein kinases were particularly well represented in our phosphoprotein dataset.

As for the quantitative analyses, our data showed that cancer cells of different phenotypes and origin had markedly different patterns of phosphorylation, thus indicating that phosphorylation could be used to classify these cells according to phenotype. PCA and unsupervised hierarchical clustering analysis classified cell lines according to their pathology (AML, lymphoma, and multiple myeloma), thus indicating that the phosphoproteomes of cells of the same pathology are more similar than those derived from different diseases. Furthermore, PC1 in the PCA and unsupervised hierarchical clustering clearly separated AML cells that have a myeloid lineage from the other two diseases that have a lymphoid lineage.

We evaluated the potential ability of phosphoproteomics data in predicting drug response through a robust cross-validation framework based on Lasso regression analysis, showing that the phosphorylation levels of a relatively small subset of phosphopeptides was informative with regard to treatment outcomes (Figure 3). In addition, using a complementary approach based on a correlation study, we identified phosphopeptide sets whose combined intensities correlated with the responses to the three compounds with remarkable correlation coefficients (R = 0.99, 0.95, and 0.94 for the responses to PI-103, MEK-i, and JAK-i, respectively). In addition to observing strong correlations with cell viability, we also found that the magnitude of changes between the phosphopeptide signatures of the most resistant and most sensitive cells were greater, or close, to 100-fold in each case (Figures 4F, 5D, and 6D). These large differences in phosphopeptide intensities are noteworthy and important as ideal predictors of response need to show robust differences and steep slopes in the regression function in addition to having a good correlation. Therefore, our proof-of-concept study indicates that phosphoproteomics may represent a general tool to identify biomarkers of response to kinase inhibitors and to classify cancers based on their likelihood that these respond to targeted therapies. Although some peptides that predicted responses to kinase inhibitors in AML were also associated with responses to the same inhibitors in lymphoma and multiple myeloma cells, there were subsets of phosphopeptides that predicted responses in AML cells only. This could be attributed to the marked differences in the patterns of basal phosphorylation between lymphoma, multiple myeloma, and AML cells (Figure 1) which may be a reflection of differences in the activity of kinase signaling across these diseases.

The correlation between PI3K pathway activity markers and sensitivity to PI3K compounds has been found to be weak in recent studies [49,50]. For example, no correlation between activating mutations on PIK3CA (the gene coding for the PI3K catalytic alpha isoform) with responses to PI3K inhibitors were found in cell lines [49], while a recently reported clinical trial found that just 30% of breast and gynecologic patients harboring PIK3CA mutations responded to PI3K therapies [50]. Dan *et al*. found a weak correlation between responses to PI3K inhibitors and phosphorylation of Akt (R < 0.45 and fold < 10 [49]), which is a marker of PI3K activity. Although the primary aim of this work was not to identify mechanisms of resistance, it was interesting to observe that, consistent with the data in Dan *et al. *[49] and Alcolea *et al. *[28], the levels of PI3K pathway activity markers did not correlate with the responses of cancer cells to the PI3K inhibitor (Figure 7A). Similarly, phosphorylation of STAT5A, a marker of JAK activity, did not correlate with the responses to JAK-i (Figure 7C). Interestingly, the phosphorylation sites that correlated with resistance were on pathways parallel to those being targeted; that is, on sites phosphorylated by PKC for the PI3K inhibitor (Figure 7B), thus raising the hypothesis that pathways parallel to those being targeted were contributing to resistance. The finding that in PKC and PI3K inhibitors had additive effects in decreasing the viability resistant AML cells (Additional file 10, Figure S6) is consistent with this possibility.

## Conclusion

Our data therefore suggest that the activity of pathways parallel to those being targeted by the specific inhibitors might contribute to intrinsic resistance to kinase inhibitors, as observed in cells that acquired resistance as a result to chronic exposure to compounds that target signaling nodes [13,14,28,51,52].

Our analysis identified phosphorylation motifs that on the whole correlated with resistance or sensitivity to kinase inhibitors (Figure 8) indicating that different kinases may be activated in resistant and sensitive cells. Thus, it may be proposed that, in this study, unbiased profiling of phosphorylation stratified AML cells based on their responses to signaling inhibitors (Figures 3, 4, 5, 6, 7, 8) because these responses may be dependent on a combination of active pathways (both target and parallel) in cells, rather than on the activity of the target kinase/pathway only. Therefore, the ability to quantitatively analyze protein phosphorylation without a preconception of the pathways that may be implicated in conferring sensitivity may represent an important advance for therapies that target kinase signaling.

## Materials and methods

### Cell culture

Cell lines were obtained as indicated in Additional file 1, Table S1 and Additional file 1, Table S2. All cell lines were grown in RPMI-1640 media supplemented with 10% FBS and 100 units/mL penicillin/streptomycin at 37°C in a humidified atmosphere of 5% CO_2_. For experiments, 10 x10^6 ^cells at 0.5 × 10^6 ^cells/mL were seeded 24 h before harvesting for each replicate.

### Viability assay

Cell lines were seeded in 96-well plates and treated 24 h later with 1 μM PI-103 (Calbiochem cat # 528100), 10 μM MEK inhibitor I (referred as MEK-i hereafter, Calbiochem cat # 444937), or 1 μM JAK inhibitor (referred as JAK-i hereafter, Calbiochem cat # 420099) for a further 72 h. Cell viability was assessed by MTS assay (CellTiter 96^® ^AQueousOne Solution Cell Proliferation assay, Promega Corporation, Madison, WI, USA).

### Cell lysis, digestion, and solid-phase extraction

Cells were harvested by centrifugation at 300 × g for 5 min and washed twice with ice cold Dulbecco's Phosphate Buffered Saline (DPBS) supplemented with 1 mM Na_3_VO_4 _and 1 mMNaF. Cell pellets were lyzed with 1 mL denaturing buffer (8 M urea in 20 mM HEPES pH 8.0) supplemented with phosphatase inhibitors (1 mM Na_3_VO_4_, 1 mMNaF, 1 mM β-glycerol phosphate, 1.25 mM sodium pyrophosphate) and further homogenized by sonication (three pulses of 15 s). Cell debris was removed by centrifugation (20,000 g for 5 min at 5°C), protein levels in the supernatant were quantified by Bradford analysis and an aliquot containing 500 μg of protein was diluted to a final volume of 1 mL in denaturing buffer. Cysteines were reduced and alkylated by sequential incubation with 4.1 mMdithiothreitol and 8.3 mMiodoacetamide for 15 min at room temperature in the dark. Samples were diluted to a final concentration of 2 M urea using 20 mM HEPES (pH 8.0) and proteins were digested with TLCK-Trypsin (20 TAME units/mg) for 16 h at 37°C. Trypsin was removed by centrifugation and the resultant peptide solutions desalted by solid-phase extraction with Sep-Pak C_18 _columns (Waters UK Ltd, Manchester, UK) following the manufacturer's instructions.

### Immobilized metal ion affinity chromatography (IMAC)

Phosphopeptides were enriched by IMAC using a modified protocol previously described [24]. In brief, Sep-Pak eluents were incubated for 1 h at room temperature with 300 μL of equilibrated Fe(III) coated sepharose beads used as 50% slurry in 50:50:0.1 acetonitrile:water:TFA. For equilibration, beads were washed twice with 200 mM EDTA, three times with 50:50:0.1 acetonitrile:water:TFA, incubated twice for 5 min at RT with 100 mM FeCl_3 _(same volume as beads) and washed six times with 50% ACN/0.1% TFA. Unbound peptides were discarded and beads were sequentially washed with 300 μL of 50% acetonitrile/0.1% TFA and 300 μL of 50% acetonitrile/1% TFA. Phosphopeptides were recovered by incubating the beads twice with 300 μL50% acetonitrile/1.5% ammonia water pH 11.0 for 1 min, after which the recovered solutions were pooled, acidified (by addition of 10% formic acid), dried in a speedvac, and stored at -80°C until analysis.

### Nanoflow-liquid chromatography tandem mass spectrometry (LC-MS/MS)

Phosphopeptides were resuspended in 0.1% TFA and analyzed in a LC-MS/MS system in random order to remove potential batch effects. Phosphoprotein separation was performed in a nanoflow ultra-high pressure liquid chromatography system (nanoAcquity, Waters) using a BEH 100 μm × 100 mm column (Waters) and a binary mobile phase gradient with 0.1% formic acid in LC-MS grade water (A) 0.1% formic acid in LC-MS grade acetonitrile (solution B). Gradients used were as follows: 1% B for 5 min, 1% B to 35% B over 100 min, a 5-min wash at 85% B, and a 7-min equilibration step at 1% B. The instrument delivered a flow rate of 5 μL/min (loading) and 400 nL/min (gradient elution) with an operating back pressure of about 3,000 psi. Phosphopeptides were directly eluted into an LTQ-Orbitrap XL mass spectrometer (Thermo Fisher Scientific, Hemel Hempstead, UK). This instrument acquired full scan survey spectra (m/z 350-1,600) with a resolution of 60,000 at m/z 400. A maximum of the five most abundant multiply charged ions present in each survey spectrum were automatically mass-selected in a data dependent manner, fragmented by collision-induced dissociation (CID) (normalized collision energy 35%), and analyzed in the LTQ. Thus, full-MS scans were followed by a maximum of 5 MS/MS scans (m/z 50-2,000) resulting in a maximum duty cycle of 2.5 s. For CID fragmentation multi-stage activation was enabled. Because chromatographic peaks were about 30 s at the base, these settings ensured that there were at least 10 data points per extracted ion chromatogram (XIC). In the data dependent acquisition, a dynamic exclusion was enabled with the exclusion list restricted to 500 entries, exclusion duration of 40 s and mass window of 10 ppm.

### Identification and quantitation of phosphopeptides

For phosphopeptide identification, mascot Distiller 2.3.2 was used to smoothen and centroid the MS/MS data. The processed files were searched against the human sequence library in the SwissProt database (version 2010_03 containing 23,000 entries [53]) using the Mascot search engine [54]. Searches were automated with Mascot Daemon (v2.2.2; Matrix Science, London, UK). The search parameters included the following parameters: trypsin as digestion enzyme with two missed cleavages allowed, carbamidomethyl (C) as fixed modification, and Pyro-glu (N-term), Oxidation (M), and Phospho (STY) as variable modifications. A mass tolerance of ± 7 ppm for the precursor ion and ± 800 mmu for fragment ions was allowed. Hits were considered significant when they had an Expectation value of < 0.05 (as returned by Mascot). False discovery rates were approximately 2% as determined by decoy database searches. Results from Mascot searches were deposited in to the ProteomeXchange Consortium [55] via the PRIDE partner repository [56] with the dataset identifier PXD000217 and DOI 10.6019/PXD000217. Sites of modification were reported when the Mascot delta score [57] was > 10; otherwise site assignment was deemed ambiguous. Phosphopeptides are reported in the results as gene name followed by phosphorylation site within the protein sequence and charge of the measured ion.

Phosphopeptide quantification was performed as described before [16,28]. Briefly, Pescal was used to align the chromatograms of all samples to be analyzed, to construct extracted ion chromatograms (XICs) for the first three isotopes of each phosphopeptide ion selected for quantitation, to pick the peaks to be quantified, and to measure their peak height and area. Windows of molecular mass and retention time were 7 ppm and 1.5 min, respectively. The resulting quantitative data were parsed into Excel files for further normalization and statistical analysis. Phosphopeptide intensities were normalized to the total chromatogram intensity and to the mean value across samples.

### PCA, clustering, and statistical analysis of MS data

To avoid interference from low intensity phosphopeptides that have the lowest quantification quality [16], a peak intensity cut-off value was established. Thus, only the phosphopeptides with a maximum intensity across all samples above that cut-off were included in further data analysis by principal component analysis (PCA) and hierarchical clustering, both performed using R software (v. 2.12.2). For this analysis, mass intensity data were log transformed, peptides and arrays were normalized and mean centered. Kendall's tau coefficient was used to measure the association between peptides and arrays. Finally complete linkage clustering was applied to calculate distances between clusters.

For stringent identification of differences across samples, phosphopeptides were considered differentially phosphorylated between cell types when the Bonferroni-corrected ANOVA and Tukey *P*values were < 0.01 and the fold difference was > 2. To correlate peptide phosphorylation with drug sensitivity, phosphopeptide intensities derived from each cell line replicate were correlated with their viability after drug treatment at either 1 μM or 10 μM relative to DMSO-treated control. The correlation between relative phosphopeptide intensity and viability was calculated using Pearson's coefficient (R) of either linear or logarithm transformed phosphorylation data.

### LASSO regression analysis

Least absolute shrinkage and selection operator (Lasso) regression is a multivariate variable selection technique with a penalization approach that controls the number of regressors to be included in the optimized model [58]. This is achieved by including a 'shrinkage' parameter *λ *in the cost function.

For each of the three drugs we considered a *n *x *p*matrix **X **(where *n *= number of biological samples (21 in this study) and *p *= number of quantified phosphopeptides (2,135 in this study)) containing the column-wise normalized (*μ *= 0, *δ *= 1) intensities of all the phosphopeptides measured across the seven cell lines (three measurements for each cell line).

We assessed the potential of these data in predicting the level of resistance/sensitivity of the seven cell lines to three kinase inhibitors through a 'leave-one-cell-line-out' approach based on lasso linear regression. With this method, seven different models were trained for each drug, leaving out the three samples corresponding to each of the seven cell lines, in turn. Then the trained models were used to make predictions on the samples that were left out in the corresponding training phase. The predictive ability was evaluated in terms of correlation between predicted and observed percentage of surviving cells when compared to the negative control, after the drug treatment.

For a given drug *D*, and a cell line *C *left out from the training phase, let **Y **be the vector containing the 18 viability scores (respectively for the remaining 18 samples) and *λ *a non-negative number. We solved through lasso regression the problem:

(1) $${\text{min}}_{\beta_{0},\text{B}}\left\{\frac{1}{2n}\sum_{i=1}^{n}\left(y_{i}-\beta_{0}-{\text{x}}_{i}^{T}B\right)+\lambda \sum_{j=1}^{p}\left|\beta_{j}\right|\right\}$$

where *n *is the number of observations (that is, the 18 samples from measurements on the remaining six cell lines, in triplicate);*y_i _*is the viability score of sample *i *following treatment with *D*; x_*i *_is the row vector containing the normalized intensities of the p phosphopeptides when measured in the *i*-th sample; *β_0 _*and ***B ***are a scalar and a p-vector, respectively. ***B ***contains the coefficients of the regressors (that is, all the phosphopeptides) to be optimized. As *λ *increases, the number of non-zero components (hence phosphopeptides with non-null coefficient in the model) decreases.

We determined the optimal value for the *λ *parameter with a three-fold cross-validation on the remaining 18 samples and solved equation (1) for vector ***B ***without considering the samples of the left out cell line. In order to reduce the instability of the final models across the three-fold cross-validation used to determine *λ*, these two final steps were repeated 20 times (for each left-out cell line) and the entries of the resulting ***B ***vector averaged across these 20 iterations, ending up in the final average model *M**_D, C _***(that is, final model for drug *D*, leaving out the cell line *C *samples). The frequency of observing a non-null coefficient for each regressor across the 20 iterations (quantifying how much the corresponding phosphopeptide is stably included in the optimal models) was also computed and reported in the final results. The viability of each left-out cell line *C *was finally predicted through the corresponding *M**_D, C_***.

In order to make the values predicted through by *M**_D, C _***on the left-out samples across the seven different cell lines *C *and the three drugs *D *comparable to each other, these values were normalized (*μ *= 0, *δ *= 1) together with the predictions of *M**_D, C _***on the corresponding training set. For the same reason, to produce the scatter plot in Figure 3, all the observed viability were normalized (*μ *= 0, *δ *= 1) drug-wisely.

To produce a final descriptive model M_D* _of response to drug *D*, the coefficients of all the phosphopeptides (and their non-null coefficient frequencies) were averaged across the seven corresponding *M**_D, C_***.

Phosphopeptides whose average non-null coefficient frequency is > 50% in these final descriptive models are those reported in the insets of Figure 3.

### Bioinformatics

Proteins containing phosphopeptides that significantly correlated with phenotypes were used for gene ontology (GO) and pathway enrichment analysis using either an in-house script that matched ontologies listed in SwissProt to each gene product or by David analysis tools [35]. As for phosphorylation motifs analysis, polypeptide sequences were obtained from each phosphopeptide in the dataset by leaving the phosphorylated residue in the center of a sequence that was flanked by seven amino acids on each side. In cases where the phosphorylated residue in the original phosphopeptide had less than seven amino acids at either terminus, these were extended by blasting them against the SwissProt database. Phosphorylation motifs were obtained from Motif-X [40] and from the literature [41] to assemble a total of 108 different motifs. Because no differences between the rates at which Ser/Thr kinases phosphorylate Ser and Thr residues have been reported, no distinction was made between p-Ser and p-Thr containing motifs. Peptides phosphorylated at tyrosines were grouped in a single motif. Polypeptide sequences in the dataset were matched to these phosphorylation motifs and the average of the normalized and log-transformed intensities of all the phosphopeptides containing each of the pre-defined phosphorylation motifs were then averaged and correlated to sensitivity. A script in VBA was written to automate the implementation of these algorithms.

### Western blot

AML cell lines were seeded at 5 × 10^5 ^cells/mL. Cells were harvested by centrifugation at 300 × g for 5 min, washed twice with ice cold Dulbecco's Phosphate Buffered Saline (DPBS), supplemented with 1 mM Na_3_VO_4 _and 1 mMNaF. Cell pellets were lyzed with lysis buffer (50 mMtris-HCL pH 7.4, 150 NaCL, 1 mM EDTA, 1% Triton X-100. Protein concentration was calculated using Bradford and 50 μg of protein were run in 10% SDS-PAGE. Proteins were transferred to PVDF membranes that were block with TBS-Tween (0.1%) supplemented with 5% skim milk. Then membranes were incubated o/n with primary antibody and secondary antibody for 1 h. Protein bands were detected using ECL. Primary antibodies pMARKS S153/156 (Cell Signaling: Cat.2741) and Vinculin (Cell Signaling: Cat. 4650) were used at 1:1,000 and 1:10,000 dilution, respectively. Secondary antibodies were used at 1:5,000 dilution.

### Data availability

Mass spectrometry data have been deposited to the ProteomeXchange with identifier PXD000217.

## Abbreviations

AML: acute myeloid leukemia; GO: gene ontology; IMAC: immobilized metal affinity chromatography; PCA:principal component analysis; XIC: extracted ion chromatogram.

## Authors' contributions

PC performed research, analyzed data, and wrote the paper; MPA performed research; FI analyzed data and wrote the paper; JCRP analyzed data; BV contributed reagents and edited the paper; JSR analyzed data; SJ contributed reagents and edited paper; PRC designed research, analyzed data, and wrote the paper.

BV is an Advisor to GSK (Stevenage, UK) and Activiomics (London, UK). PRC is an Advisor to Activiomics (London, UK).

All authors read and approved the final manuscript.

## Supplementary Material

Additional file 1 **Table S1 - Hematological cell lines used to compare phosphoproteomes of different hematological cancers**. Table S2 - AML cell lines used to correlate sensitivity to kinase inhibitors with phosphoproteomics data. Click here for file

Additional file 2 **Figure S1 - Workflow and distribution of the identified phosphorylation sites**. Click here for file

Additional file 3 **Dataset 1 - Identities and quantitative values of all phosphopeptides identified in AML, lymphoma, and multiple myeloma cell lines**. Click here for file

Additionla file 4 **Figure S2 - Protein classes represented in the phosphoproteomes of hematological cancer cell lines**. Click here for file

Additional file 5 **Figure S3 - Representative examples of phosphopeptides differentially regulated in AML, lymphoma, and multiple myeloma cell lines**. Click here for file

Additional file 6 **Dataset 2 - Correlation of phosphoprotein data with responses to kinase inhibitors in AML**. Click here for file

Additional file 7 **Figure S4 - Scatter plots between predicted/observed viability scores for individual drugs with cell lines identifiers, correlations scores, and *P *values**. Click here for file

Additional file 8 **Dataset 3 - Final descriptive models of drug responses as resulting from the lasso regression analysis**. Listed are predictive phosphopeptides together with their average coefficients and inclusion frequency. Click here for file

Additional file 9 **Figure S5 - Association between the markers of sensitivity to kinase inhibitors found for AML cells with the sensitivity to the same inhibitors in lymphoma and multiple myeloma cells**. Click here for file

Additional file 10 **Figure S6 - Pathway analysis of phosphopeptides that correlate with the responses to PI-103**. Click here for file

Additional file 11 **Figure S7 - An inhibitor of PKC reduced the viability of AML cells resistant to PI-103 inhibition and had an additive effect with PI-103**. Click here for file
