# Supplementary material for: Phosphoproteomics data classify hematological cancer cell lines according to tumor type and sensitivity to kinase inhibitors
Source: Genome Biol. 2013 Apr 29;14(4):R37. doi: 10.1186/gb-2013-14-4-r37 (PMC4054101; doi:10.1186/gb-2013-14-4-r37)
Supplement: Additional file 1 — Table S1 - Hematological cell lines used to compare phosphoproteomes of different hematological cancers. Table S2 - AML cell lines used to correlate sensitivity to kinase inhibitors with phosphoproteomics data. [file gb-2013-14-4-r37-S1.DOCX]

**Table S1. Hematological cell lines used to compare phosphoproteomes of different hematological cancers**

| Cell Line | Collection | Reference | Features |
| --- | --- | --- | --- |
| P31/Fuj | JCRB | JCRB 0091 | AML |
| CTS | Dr T. Sato | Ref [^9^](#_ENREF_9) | AML |
| MV4-11 | DSMZ | ACC 102 | AML |
| RL | ATTC | CRL-2261 | B-Cell Lymphoma (Non-Hodgkin) |
| SU-DHL-6 | ATTC | CRL-2959 | B-Cell Lymphoma (Non-Hodgkin) |
| DoHH2 | DSMZ | ACC 47 | B-Cell Lymphoma (Non-Hodgkin) |
| RPMI-8226 | ATTC | CCL-155 | Multiple Myeloma |
| U266B1 | ATTC | TIB-196 | Multiple Myeloma |
| OMP2 | DSMZ | ACC 50 | Multiple Myeloma |

**Table S2. AML cell lines used to correlate sensitivity to kinase inhibitors with phosphoproteomics data**

| Cell Line | Collection | Reference |
| --- | --- | --- |
| P31/Fuj | JCRB | JCRB 0091 |
| HEL | JCRB | JCRB 0062 |
| MV4-11 | DSMZ | ACC 102 |
| KG1 | JCRB | JCRB 0065 |
| Kasumi-1 | JCRB | JCRB 1003 |
| CMK-86 | JCRB | IFO50428 |
| AML-193 | ATCC | CRL-9589 |
