# Supplementary material for: Phosphoproteomics data classify hematological cancer cell lines according to tumor type and sensitivity to kinase inhibitors
Source: Genome Biol. 2013 Apr 29;14(4):R37. doi: 10.1186/gb-2013-14-4-r37 (PMC4054101; doi:10.1186/gb-2013-14-4-r37)
Supplement: Additional file 2 — Figure S1 - Workflow and distribution of the identified phosphorylation sites. [file gb-2013-14-4-r37-S2.DOC]

Lysis

of Cells

Trypsin

digestion

Desalting

IMAC

phosphoenrichment

LC

-

MS/MS (Orbitrap)

Data analysis

Identification :

Mascot

Quantification:

Pescal

2050 phosphopeptides

in

1664 Proteins

(a)

(b)

1254

74%

344

20%

98

6%

pSer

pThr

pTyr

**Figure S1. Workflow and distribution of the identified phosphorylation sites.** (a) Following cell lysis and protein digestion with trypsin, the resulting peptides were desalted, subjected to IMAC phospho-enrichment and run in an LC-MS/MS system. MS data were analyzed with MASCOT to identify the peptides present in the samples and with PESCAL to quantify them. Further data analysis and mining was performed using different statistical tools. (b) Distribution of the three types of phosphorylation sites across all phosphopeptides indentified.
