# Supplementary material for: Phosphoproteomics data classify hematological cancer cell lines according to tumor type and sensitivity to kinase inhibitors
Source: Genome Biol. 2013 Apr 29;14(4):R37. doi: 10.1186/gb-2013-14-4-r37 (PMC4054101; doi:10.1186/gb-2013-14-4-r37)
Supplement: Additionla file 4 — Figure S2 - Protein classes represented in the phosphoproteomes of hematological cancer cell lines. [file gb-2013-14-4-r37-S4.DOC]

**Figure S2. Protein classes represented in the phosphoproteomes of hematological cancer cell lines.** Gene ontology (GO) distributions between the phosphoproteins detected in hematological cell lines were compared to the proteins listed in the SwissProt database. (A) Comparison of GO distributions comprised in the cellular component domain. (B) Comparison of GO distributions comprised in the cellular process domain. (C) Comparison of GO distributions comprised in the molecular function domain.
