# Supplementary material for: Phosphoproteomics data classify hematological cancer cell lines according to tumor type and sensitivity to kinase inhibitors
Source: Genome Biol. 2013 Apr 29;14(4):R37. doi: 10.1186/gb-2013-14-4-r37 (PMC4054101; doi:10.1186/gb-2013-14-4-r37)
Supplement: Additional file 5 — Figure S3 - Representative examples of phosphopeptides differentially regulated in AML, lymphoma, and multiple myeloma cell lines. [file gb-2013-14-4-r37-S5.DOC]

**Figure S3. Representative examples of phosphopeptides differentially regulated in AML, lymphoma and multiple myeloma cell lines.** The data outlined in Figure 1 were mined to identify specific phosphopeptides differentially regulated in each pathological group. Phosphopeptides are named as gene name from which they derive followed by the phosphorylation site and charge. Anova values were corrected for multiple testing using the Bonferroni method. Fold 1 indicates fold change between AML and lymphoma calculated as average intensity in AML samples divided by average intensity in lymphoma samples, Fold 2 indicates fold change between AML and multiple myeloma and Fold 3 indicates fold change between lymphoma and multiple myeloma.
