# Supplementary material for: Phosphoproteomics data classify hematological cancer cell lines according to tumor type and sensitivity to kinase inhibitors
Source: Genome Biol. 2013 Apr 29;14(4):R37. doi: 10.1186/gb-2013-14-4-r37 (PMC4054101; doi:10.1186/gb-2013-14-4-r37)
Supplement: Additional file 7 — Figure S4 - Scatter plots between predicted/observed viability scores for individual drugs with cell lines identifiers, correlations scores, and P values. [file gb-2013-14-4-r37-S7.DOC]

Figure S4. Scatter plots between predicted/observed viability scores for individual drugs with cell lines identifiers, correlations scores and p-values
