# Supplementary material for: Phosphoproteomics data classify hematological cancer cell lines according to tumor type and sensitivity to kinase inhibitors
Source: Genome Biol. 2013 Apr 29;14(4):R37. doi: 10.1186/gb-2013-14-4-r37 (PMC4054101; doi:10.1186/gb-2013-14-4-r37)
Supplement: Additional file 9 — Figure S5 - Association between the markers of sensitivity to kinase inhibitors found for AML cells with the sensitivity to the same inhibitors in lymphoma and multiple myeloma cells. [file gb-2013-14-4-r37-S9.DOC]

**Figure S5. Association between the markers of sensitivity to kinase inhibitors found for AML cells with the sensitivity to the same inhibitors in lymphoma and multiple myeloma cells.** (a) Responses of lymphoma and multiple myeloma and cells to PI-103, JAK-i and MEK-i. Correlation between the intensities of phosphorylation sites shown and cell viability as a function to treatment with PI-103 (b), JAK-i (c) and MEK-i (d).
