# Supplementary material for: Phosphoproteomics data classify hematological cancer cell lines according to tumor type and sensitivity to kinase inhibitors
Source: Genome Biol. 2013 Apr 29;14(4):R37. doi: 10.1186/gb-2013-14-4-r37 (PMC4054101; doi:10.1186/gb-2013-14-4-r37)
Supplement: Additional file 10 — Figure S6 - Pathway analysis of phosphopeptides that correlate with the responses to PI-103. [file gb-2013-14-4-r37-S10.DOC]

**Figure S6. Pathway analysis of phosphopeptides that correlate with the responses to PI-103**

The accession numbers of phosphoproteins that correlated with resistance or sensitivity to PI-103 were loaded in DAVID pathway analysis tool. (a) The pathway with greater score for phosphopeptides that correlated with resistance was for protein kinase C signaling. (b) Protein kinases were also well represented. (d) Proteins with the gene ontology of transcription correlated with sensitivity to PI-103 or (d) with resistance.
