# Supplementary material for: Phosphoproteomics data classify hematological cancer cell lines according to tumor type and sensitivity to kinase inhibitors
Source: Genome Biol. 2013 Apr 29;14(4):R37. doi: 10.1186/gb-2013-14-4-r37 (PMC4054101; doi:10.1186/gb-2013-14-4-r37)
Supplement: Additional file 11 — Figure S7 - An inhibitor of PKC reduced the viability of AML cells resistant to PI-103 inhibition and had an additive effect with PI-103. [file gb-2013-14-4-r37-S11.DOC]

**Figure S7. An inhibitor of PKC reduced the viability of AML cells resistant to PI-103 inhibition and had an additive effect with PI-103.** (a) The AML cell lines P31/Fuj, HEL and MV4-11 were treated with the indicated concentrations of the PI3K/mTOR inhibitor PI-103 and the PKC inhibitor Go6976 for 72h, and cell viability measured by MTS. (b) P31/Fuj, HEL and MV4-11 cells were treated with the indicated concentrations of Go6976 for 72h and cell viability measured using MTS. (c) P31/Fuj, HEL and MV4-11 were harvested and levels of MARKS phosphorylated at serines 156 and 156 where analyzed by Western blot, bands were quantified measuring optic densities.
